# Ischemic Postconditioning Reduces Reperfusion Arrhythmias by Adenosine Receptors and Protein Kinase C Activation but Is Independent of K_ATP_ Channels or Connexin 43

**DOI:** 10.3390/ijms20235927

**Published:** 2019-11-25

**Authors:** Emiliano Raúl Diez, Jose Antonio Sánchez, Natalia Jorgelina Prado, Amira Zulma Ponce Zumino, David García-Dorado, Roberto Miguel Miatello, Antonio Rodríguez-Sinovas

**Affiliations:** 1 Facultad de Ciencias Médicas, Universidad Nacional de Cuyo, Mendoza 5500, Argentina; diez.emiliano@fcm.uncu.edu.ar (E.R.D.); nprado@mendoza-conicet.gob.ar (N.J.P.); aponce@fcm.uncu.edu.ar (A.Z.P.Z.); rmiatell@fcm.uncu.edu.ar (R.M.M.); 2 Institute of Medical and Experimental Biology of Cuyo, CONICET, Mendoza 5500, Argentina; 3 Cardiovascular Diseases Research Group, Department of Cardiology, Vall d’Hebron University Hospital and Research Institute, Universitat Autònoma de Barcelona, Departament de Medicina, Pg. Vall d’Hebron 119-129, 08035 Barcelona, Spain; joseant.msq@gmail.com (J.A.S.); dgdorado@vhebron.net (D.G.-D.); 4 Centro de Investigación Biomédica en Red sobre Enfermedades Cardiovasculares (CIBERCV), 28029 Madrid, Spain

**Keywords:** arrhythmia, postconditioning, adenosine receptors, PKC, KATP channels, connexin 43

## Abstract

Ischemic postconditioning (IPoC) reduces reperfusion arrhythmias but the antiarrhythmic mechanisms remain unknown. The aim of this study was to analyze IPoC electrophysiological effects and the role played by adenosine A_1_, A_2A_ and A_3_ receptors, protein kinase C, ATP-dependent potassium (K_ATP_) channels, and connexin 43. IPoC reduced reperfusion arrhythmias (mainly sustained ventricular fibrillation) in isolated rat hearts, an effect associated with a transient delay in epicardial electrical activation, and with action potential shortening. Electrical impedance measurements and Lucifer-Yellow diffusion assays agreed with such activation delay. However, this delay persisted during IPoC in isolated mouse hearts in which connexin 43 was replaced by connexin 32 and in mice with conditional deletion of connexin 43. Adenosine A_1_, A_2A_ and A_3_ receptor blockade antagonized the antiarrhythmic effect of IPoC and the associated action potential shortening, whereas exogenous adenosine reduced reperfusion arrhythmias and shortened action potential duration. Protein kinase C inhibition by chelerythrine abolished the protective effect of IPoC but did not modify the effects on action potential duration. On the other hand, glibenclamide, a K_ATP_ inhibitor, antagonized the action potential shortening but did not interfere with the antiarrhythmic effect. The antiarrhythmic mechanisms of IPoC involve adenosine receptor activation and are associated with action potential shortening. However, this action potential shortening is not essential for protection, as it persisted during protein kinase C inhibition, a maneuver that abolished IPoC protection. Furthermore, glibenclamide induced the opposite effects. In addition, IPoC delays electrical activation and electrical impedance recovery during reperfusion, but these effects are independent of connexin 43.

## 1. Introduction

Severe ventricular arrhythmias are potentially lethal events during acute myocardial infarction [1]. However, sinus rhythm restoration by electrical or pharmacological antiarrhythmic therapies could be challenging or unavailable [2]. Reperfusion therapies are crucial during acute myocardial ischemia. Unfortunately, an adverse effect of these therapies is the so-called reperfusion injury, that involves myocardial cell damage/death, mechanical and microvascular dysfunction, and arrhythmias [3,4]. Reperfusion arrhythmias are an early marker of myocardial damage [5]. The occurrence of ventricular tachycardia or fibrillation during primary angioplasty is a serious complication in acute myocardial infarction patients, and is associated with higher mortality [6,7,8]. 

Ischemic postconditioning (IPoC) reduces the incidence and severity of reperfusion arrhythmias. Brief episode(s) of ischemia during reperfusion can prevent arrhythmia development if applied at the beginning of reperfusion and can also restore sinus rhythm if applied later [9,10]. Antiarrhythmic protection persists in animal models of senescence and hypertension [11,12]. Furthermore, IPoC reduces reperfusion arrhythmias and the dispersion of the QT interval in patients [13,14]. However, the mechanisms involved in the antiarrhythmic effect of IPoC remain elusive. Sarcolemmal and mitochondrial K_ATP_ channel inhibitors did not modify protection against reperfusion ventricular tachycardia and fibrillation in dogs submitted to IPoC [15]. Dow et al. showed that the IPoC antiarrhythmic effect is not affected by treatment with either a non-selective adenosine receptor blocker, an inhibitor of the mitochondrial K_ATP_ channels, or an inhibitor of the phosphoinositide-3-kinase, administered before index regional ischemia [16]. Kolettis et al. reported a prominent antiarrhythmic effect of IPoC in the in vivo rat model of ischemia-reperfusion, but they were not able to observe any change in ECG recordings, heart rate variability indices, or monophasic action potential characteristics that could give an indication of the underling mechanisms [17]. Recently, Spannbauer et al. found that IPoC induces a recovery of the R-amplitude, ST-segment elevation, and QTc interval, and decreases arrhythmias during reperfusion [4].

This work aims to assess the effects of IPoC on transmembrane action potential characteristics and electrical activation and to analyze the mechanims involved in these effects, including the role played by gap junctional communication through connexin 43 (Cx43), adenosine receptors, using subtype-selective receptor blockers, and that of protein kinase C activation and K_ATP_ channels.

## 2. Results

### 2.1. Electrophysiological Effects of IPoC in Isolated Rat Hearts Submitted to Regional Ischemia

**Figure 1:**
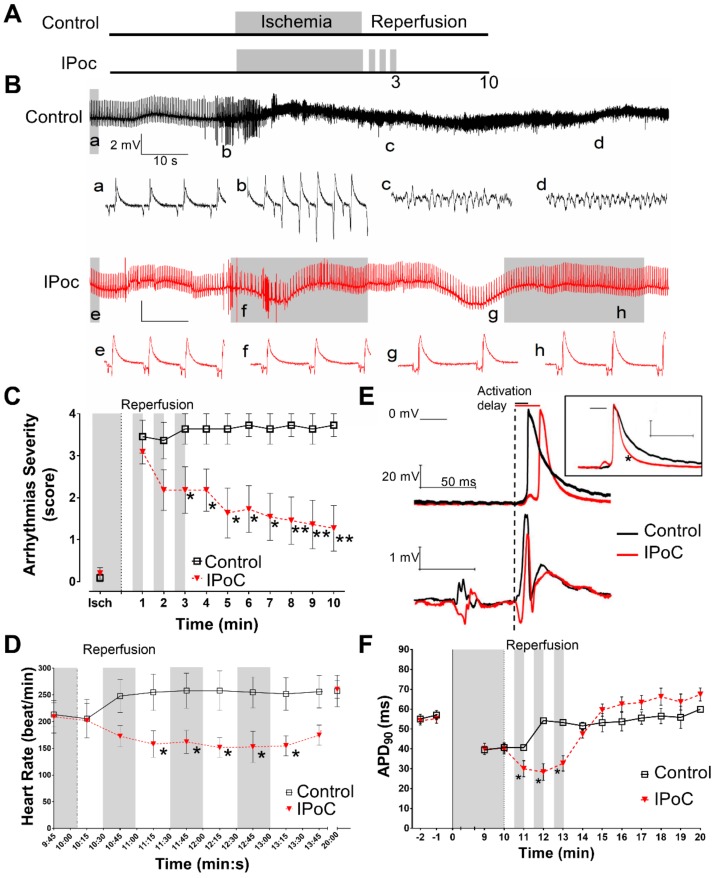
Electrophysiological effects of IPoC in isolated rat hearts. (**A**) Experimental protocol: 10 min of regional myocardial ischemia (indicated in grey) followed by 10 min reperfusion as Control; IPoC by 3 cycles of reperfusion/regional ischemia, 30 s each. (**B**) Representatives ECG from the first 2 min of reperfusion. The Control heart developed ventricular fibrillation and IPoC suffered transient episodes of ventricular tachycardia and bradycardia. Lower case letters from a to h corresponds to 1 s traces showed below. (**C**) The hearts did not develop sustained arrhythmias prior reperfusion. Control group presented severe ventricular arrhythmias through reperfusion whereas IPoC progressively reduced the severity. (**D**) IPoC induced transient bradycardia. (**E**) Representative transmembrane potential and ECG simultaneously obtained during the 2nd min of reperfusion. Dashed vertical line indicates the beginning of the QRS complex used to measure the delay to epicardial activation. In the inset, the action potentials were artificially aligned to 0 phase for better identification of action potential duration (APD) shortening. (**F**) Both groups have similar duration prior to reperfusion reaching values around 40 ms at the end of ischemia. During reperfusion, Control hearts recovered preischemic APD_90_ values. IPoC induced a transient shortening during the first 3 min of reperfusion. * *p* < 0.05 and ** *p* < 0.01 for Control vs. IPoC by repeated measures ANOVA.

IPoC (3 cycles of 30 s of reperfusion and 30 s of regional ischemia) reduced the severity of reperfusion arrhythmia in isolated rat hearts when applied after 10 min of regional ischemia (Figure 1). Ventricular fibrillation incidence decreased from 12/15 in control hearts to 4/16 in IPoC (*p* = 0.0038, Fisher’s exact test). IPoC also reduced ventricular fibrillation duration (Table 1). Moreover, bradycardia was predominant in those hearts from IPoC group that were in sinus rhythm during the first three minutes of reperfusion (Figure 1D).

IPoC induced a significant delay in action potential upstroke respect to the onset of the QRS complex as compared with control hearts (21.5 ± 1.4 vs. 13.2 ± 1.3 ms, *p* < 0.05 by repeated measures ANOVA) (Figure 1E) In addition, IPoC induced a transient action potential shortening during the maneuver (Figure 1E,F).

During reperfusion the control group showed a rapid recovery of resting membrane potential, from −63.5 ± 3.2 mV to −86.1 ± 3.1 mV in the first minute of reperfusion, and then remained stable at −81.4 ± 5.1 mV. Resting membrane potential recovery in IPoC group was slightly delayed and more gradual, from −65.3 ± 2.8 mV to −77.5 ± 5.2 mV during the first two minutes of reperfusion, and then stabilized at −79.5 ± 3.2 mV. Action potential amplitude was maintained at 87.6 ± 8.2 and 89.8 ± 10.1 mV during reperfusion in control and in IPoC hearts, respectively.

**Figure 2:**
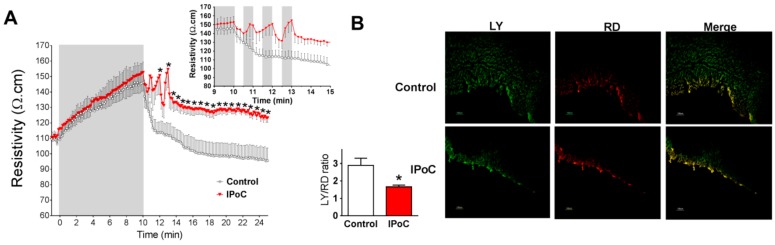
Myocardial resistivity and chemical communication through gap junction in isolated rat hearts. (**A**) Resistivity increases during ischemia (in grey) in both groups and rapidly recovered during reperfusion in Control hearts but not in IPoC treated ones. The inset shows the values from the last min of ischemia to the 5th min of reperfusion.* *p* < 0.05 by repeated measures ANOVA (**B**). Representative images of Lucifer yellow (LY) and rodamine (RD) spread taken from the 4th min of reperfusion indicate lower diffusion through gap junction in IPoC group. Quantitative comparison of the ratio of LY/RD for each group (*n* = 4 each). * *p* < 0.05 Control vs. IPoC by Mann-Whitney U test.

Myocardial impedance recordings showed a marked increase in tissue resistivity during ischemia (Figure 2A). Control group showed a fast recovery upon reperfusion. IPoC delayed the recovery of tissue resistivity and maintained it persistently higher during reperfusion (Figure 2A).

IPoC reduced chemical communication through gap junctions, as assessed by Lucifer yellow (LY) diffusion (Figure 2B). In contrast, area stained with rhodamine (RD), which is not permeable through gap junctions, and indicates diffusion thought cells with broken sarcolemma, was similar in both groups. Accordingly, the ratio LY/RD was significantly reduced in IPoC group (Figure 2B).

### 2.2. Connexin 43 Is Not Essential for IPoC Effects in Isolated Mice Hearts Submitted to Global Myocardial Ischemia

**Figure 3:**
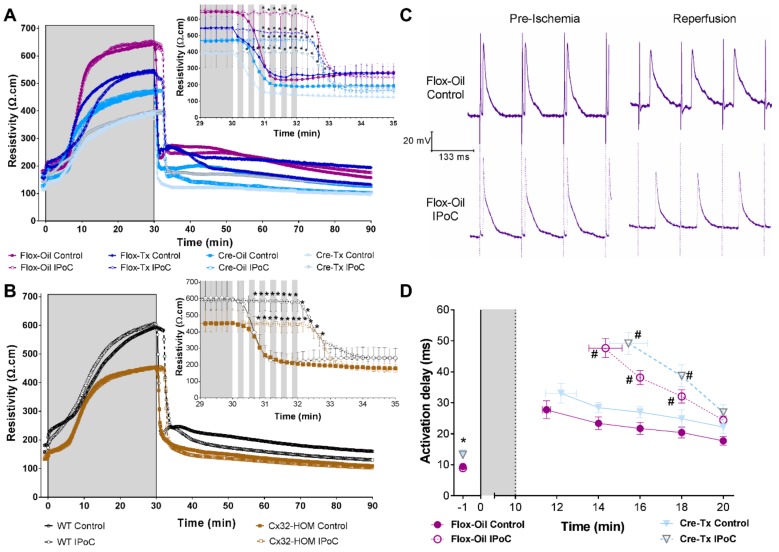
IPoC delayed the recovery of tissue resistivity and epicardial activation regardless of Cx43 expression in isolated mice hearts. (**A**) After 30 min of global ischemia (in grey), 6 cycles of 10 s reperfusion/10s global ischemia delayed tissue resistivity recovery in Cx43^fl/fl^ (Flox) and in Cx43^Cre-ER(T)/fl^ (Cre) mice treated with either oil (Flox-Oil 100%, and Cre-Oil 50% Cx43 expression) or 4-Hydroxytamoxifen (Tx) (Flox-Tx 100%, and Cre-Tx <5% Cx43 expression). The inset shows the values from the last min of ischemia to the 5th min of reperfusion. * *p* < 0.05 vs. each Control by repeated measures ANOVA. (**B**) IPoC delayed tissue resistivity recovery in mice in which the coding region of Cx43 is replaced by that of connexin 32 (Cx32). Only homozygous (Cx32-HOM) and wild-type (WT) animals were included. (**C**) IPoC induced a delay in epicardial activation and action potential shortening as can be seen in representative traces from the 4th min of reperfusion. (**D**) Cre-Tx showed a delay before ischemia but the response to IPoC was no different from those with full content of Cx43 (Flox-Oil). * *p* < 0.05 vs. Flox and # *p* < 0.05 vs. Control by repeated measures ANOVA.

Similar to isolated rat hearts, tissue resistivity markedly increased during global ischemia in isolated mice hearts, and rapidly returned to preischemic values during reperfusion (compare Figure 2A and Figure 3A,B ). IPoC delayed tissue resistivity recovery regardless of the content of connexin 43 present in the heart of Cx43^Cre-ER(T)/fl^ mice treated with either oil (50% Cx43 expression) or 4-hydroxytamoxifen (Cre-Tx, <5% Cx43 expression) (Figure 3A) or the connexin isoform (homozygosity for connexin 43 replacement by connexin 32, Cx32-HOM; or wildtype for connexin 43, WT) (Figure 3B). Moreover, IPoC induced similar activation delays and prolonged the time until recapture of the pacemaker, independently of the level of connexin 43 expression (Figure 3C,D). Activation delays diminished progressively until minute 10th of reperfusion.

**Figure 4:**
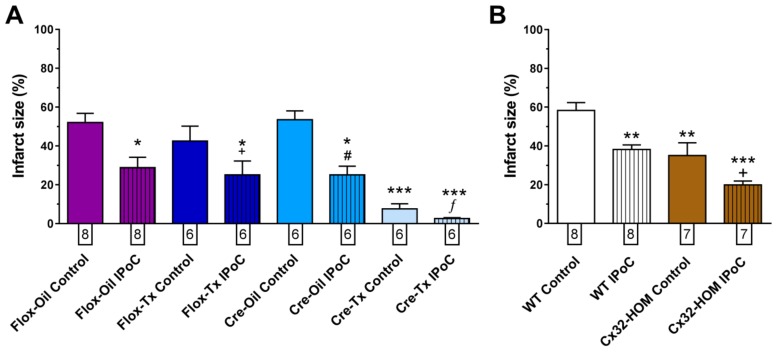
Infarct size reduction by IPoC persisted in isolated mice hearts with modified connexin expression. (**A**) IPoC reduced infarct size in Flox and Cre mice. Numbers below each column indicate the number of hearts measured. * *p* < 0.05 and *** *p* < 0.001 vs. Flox-Oil Control +, #, ƒ *p* < 0.05 vs. each Control by two-way ANOVA (**B**). IPoC reduced infarct size in WT and Cx32-HOM mice. Numbers below each column indicate the number of hearts measured. ** *p* < 0.01 and *** *p* < 0.001 vs. WT Control and + *p* < 0.05 vs. Cx32-HOM Control by two-way ANOVA.

As sustained ventricular tachyarrhythmias induction by ischemia/reperfusion is difficult in isolated mice hearts, we determined the effectiveness of our IPoC maneuver by assessing their effect on infarct size. IPoC reduced the extent of infarction, even in those hearts that were relatively protected by its genetic background (Figure 4).

### 2.3. Adenosine Receptors and PKC Activations, but Not K_ATP_ Channels, Are Needed for IPoC Antiarrhythmic Protection

**Figure 5:**
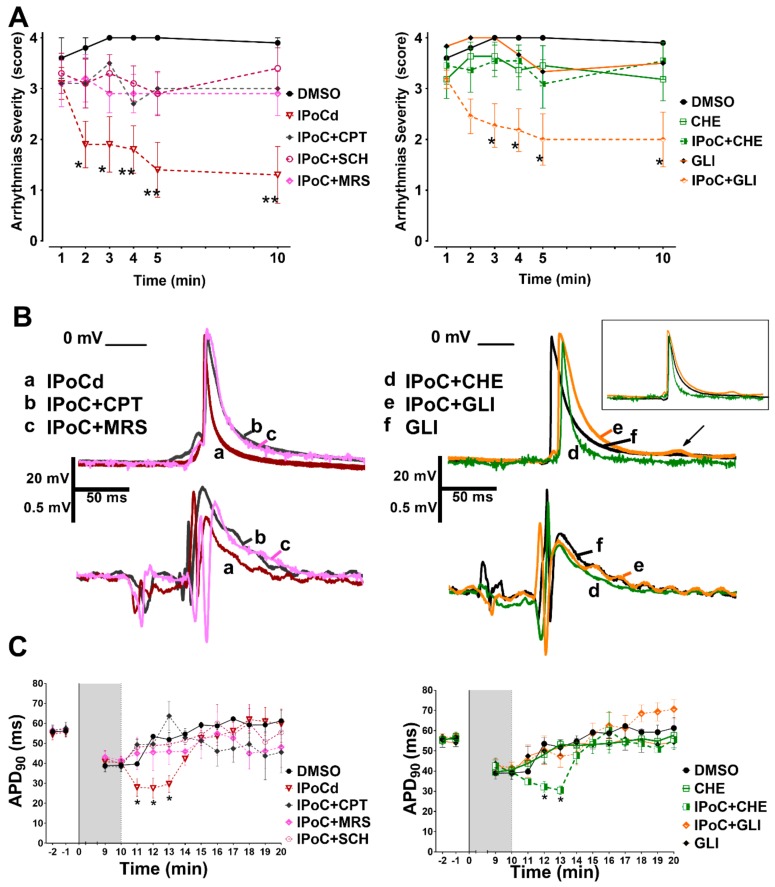
Adenosine receptors and PKC activations are needed to IPoC protection, but not K_ATP_ channels. (**A**) Since some of the drugs were dissolved in dimetilsophoxide (DMSO) also postconditioning was performed under this condition (IPoCd). Arrhythmias’ severity during reperfusion was reduced by IPoCd and all co-administered treatments abrogated protection except glibenclamide (IPoC + GLI). (**B**) Adenosine receptors inhibitors and glibenclamide, but not chelerythrine, prevented action potential shortening induced by IPoCd. In the inset, the action potentials were artificially aligned to 0 phase for better contrast of APD. The arrow indicates a delayed afterdepolarization. (**C**) Adenosine receptors blockade and glibenclamide prevented the transient shortening induced by IPoCd during the first 3 min of reperfusion, but CHE did not affect APD shortening.

Adenosine A_1_, A_2A_ and A_3_ receptor blockade blunted the antiarrhythmic effect of IPoC in isolated rat hearts submitted to regional ischemia (Figure 5A). The transient bradycardia induced by IPoC persisted despite adenosine receptors blockade (IPoC+CPT 171.4 ± 17.9 beat/min, IPoC+SCH 173.9 ± 16.8 beat/min, IPoC+MRS 153.4 ± 19.2 beat/min). In contrast, adenosine receptor blockade blunted the action potential shortening induced by IPoC (Figure 5B,C).

PKC inhibition with chelerythrine (CHE) abrogated the antiarrhythmic effect of IPoC but did not modify APD shortening induced by IPoC (Figure 5). In contrast, selective K_ATP_ channels inhibition with glibenclamide (GLI) did not interfere with the antiarrhythmic effect of IPoC but blocked the APD shortening induced by IPoC (Figure 5). GLI group showed a reduction in ventricular fibrillation duration but severity persisted high due to episodes of ventricular tachycardia (Table 1 and Figure 5). Both ventricular arrhythmias were reduced in IPoC + GLI group. Bradycardia during IPoC continued in the presence of CHE and GLI (IPoC + CHE 175.4 ± 17.3 beat/min, and IPoC + GLI 163.9 ± 20.1 beat/min).

The action potential amplitude and the resting membrane potential did not change with any of the five inhibitors when administered alone or simultaneously to IPoC, as can be appreciated in Figure 5 representative traces.

### 2.4. Adenosine Intermitet (ADOi) Administration during Reperfusion Reproduced Some of the Electrophysiological Effects of IPoC

**Figure 6:**
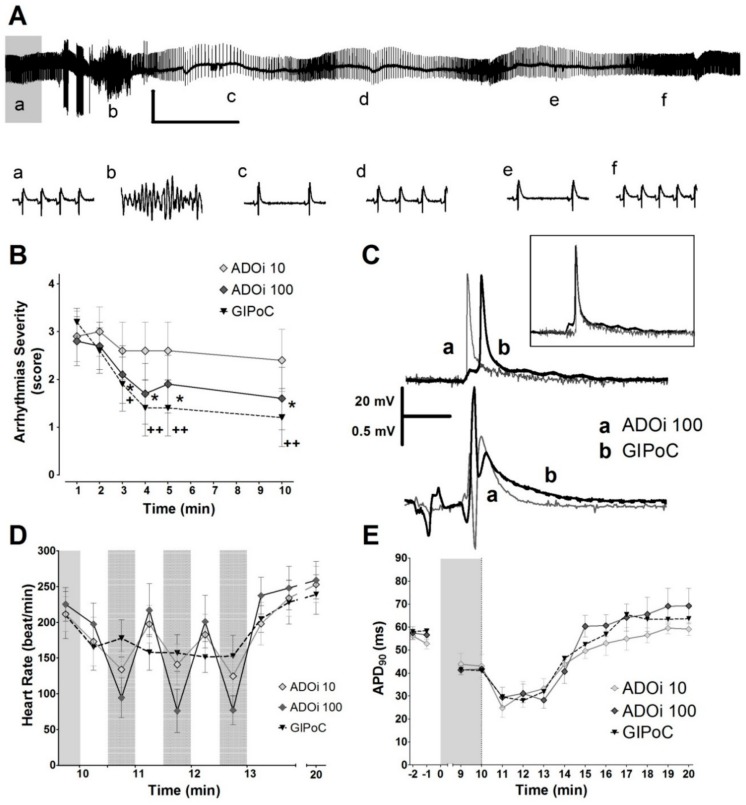
Antiarrhythmic effect and transient bradycardia induced by adenosine and GIPoC. (**A**) Representatives ECG traces from the first 3 min of reperfusion that show sinus rhythm recovery by intermittent adenosine in a heart that developed a ventricular arrhythmia. Adenosine also induced transient episodes of bradycardia. Vertical scales indicate 1 mV and horizontal 20 s. Lower case letters from a to f indicate the moment in which the corresponding 1 s traces showed below were taken. (**B**) The higher dose of adenosine almost reduced the severity of arrhythmias to the same level of protection than GIPoC, but the lower dose failed to protect. (**C**) Adenosine and GIPoC shortened action potential duration, but only GIPoC delayed the activation of the epicardial tissue previously submitted to regional ischemia. Adenosine shortened QT duration. (**D**) Adenosine and GIPoC induced transient bradycardia, followed by a progressive hear rate recovery towards the minute 10th of reperfusion. Bradycardia was more pronounced in adenosine treated hearts. Grey areas with black dots indicate global ischemia or adenosine treatment. (**E**) During the first 3 min of reperfusion, ADOi 10, ADOi 100 and GIPoC shortened the action potential duration, and then recover preischemic values. * *p* < 0.05 and ** *p* < 0.01 for Control vs. IPoC by repeated measures two-way ANOVA.

A low dose of ADOi (10 µmol/L) did not protect against reperfusion arrhythmias, but a higher dose (100 µmol/L) protected as much as global IPoC (GIPoC), and both reached a protection comparable to that obtained with regional IPoC (compare Figure 1 and Figure 6). The action potential shortened with both adenosine doses and with GIPoC. Transient bradycardia was more pronounced with ADOi than during IPoC and GIPoC (compare Figure 1D and Figure 5D). GIPoC induced action potential delay (22.1 ± 3.3 ms) but the delay was not present in ADOi groups (ADOi10 13.9 ± 2.3 ms, and ADOi100 13.8 ± 2.9 ms).

## 3. Discussion

The mechanisms involved in the antiarrhythmic effects of IPoC remained elusive until now. The present study demonstrates regional activation delay and action potential shortening that could be undetectable by QT interval measurements on the ECG (see Figure 1, Figure 5 and Figure 6). Our results indicate that IPoC antiarrhythmic protection involves adenosine receptors and PKC activation but not K_ATP_ channels or Cx43.

Selective adenosine A_1_, A_2A_ and A_3_ receptor blockade counteracts the antiarrhythmic effect of IPoC. This result differs from those previously reported by Dow et al. in in situ rat hearts, in which a non-selective adenosine blocker failed to attenuate the protection induced by IPoC against ventricular tachycardia after 5 min of regional ischemia [16]. In addition to differences in drug selectivity, these discrepancies could be related to the experimental model used. In Langendorff perfused rat hearts, reperfusion after 10 min of regional ischemia, induces ventricular tachycardia followed by sustained ventricular fibrillation. We found that adenosine receptors blockade inhibits mainly the anti-fibrillatory effect of IPoC. Also, the timing and route of the drugs administration might explain the discrepancies between both studies. We perfused adenosine blockers during the IPoC maneuver, whereas Dow et al. injected the drugs before the 5 min period of regional ischemia. Our results agree with the anti-fibrillatory effects of adenosine A_1_ and A_2_ receptor activation observed in rat hearts [18,19]. The present controversy regarding the involvement of adenosine receptors in the antiarrhythmic effect of IPoC during reperfusion extends also to studies focusing on infarct size reduction [20,21,22]. A possible limitation of our study is that we used only pharmacological tools to demonstrate an involvement of adenosine receptors in IPoC protection against reperfusion arrhythmias. Genetic approaches would complement and strengthen our hypothesis. However, severe reperfusion arrhythmias like ventricular fibrillation or sustained ventricular tachycardia are difficult to study in mice [23,24].

In addition to its antiarrhythmic effect, IPoC induces APD shortening. This effect seems not to be essential for reperfusion arrhythmia protection. Previous reports suggested that APD shortening is antiarrhythmic, due to a reduction in calcium accumulation and a consequent suppression of afterdepolarizations and triggered activity in cardiac myocytes [25,26,27]. Since all three adenosine receptor blockers antagonized the IPoC-associated APD shortening and blunted the antiarrhythmic effect, one would expect a cause-effect relationship. However, chelerythrine treatment point to the contrary, as PKC inhibition attenuated the antiarrhythmic effect of IPoC, but not the APD shortening. Indeed, the APD lengthening induced by glibenclamide during IPoC did not abrogate the antiarrhythmic effect, confirming previous reports using both selective sarcolemmal or mitochondrial K_ATP_ channel blockers [15,16]. Furthermore, reentrant arrhythmias are expected to be aggravated by APD shortening, which usually shortens the refractory period. Lack of relation between action potential morphology and cardioprotection was previously reported in preconditioning studies [28].

The activation of PKC during IPoC is necessary for its antiarrhythmic effect. Our results with chelerythrine showed that inhibition of PKC abrogates the antiarrhythmic effect of IPoC. Other authors showed that this drug also abolished IPoC infarct size limiting effect in rat hearts, and Zatta et al., demonstrated that PCK epsilon is activated by IPoC [29,30]. Activation of the PKC epsilon isoform reduces reperfusion arrhythmias [31]. It should be emphasized that multiple studies have described the involvement of PKC translocation in adenosine-mediated cardioprotection [32,33], a pathway that can be activated by other postconditioning triggers [34,35,36]. The electrophysiological effects of PKC isoform activation during IPoC remain to be established.

We found persistence of the antiarrhythmic effect of IPoC in glibenclamide-treated hearts despite the induction of APD lengthening. Several stimuli activate K_ATP_ channels, like ATP depletion, oxidative stress, low pH, and phosphorylation, leading to ADP shortening [32]. As mentioned above, APD shortening reduces triggered activity, but also could be proarrhythmic [33,37]. Our findings agree with previous data showing that glibenclamide reduces the duration of ventricular fibrillation during reperfusion in rat hearts, but ventricular tachycardia persisted during reperfusion leading to a high arrhythmia severity score (Figure 5) [38]. The latter agrees with reports showing that glibenclamide had no effect on reperfusion ventricular tachyarrhythmias [33,39]. Our results should be taken carefully because glibenclamide could impair ventricular contraction and reduce coronary blood flow, predisposing to malignant arrhythmias [40,41,42]. The persistence of antiarrhythmic protection by IPoC after glibenclamide treatment could be of clinical interest in diabetic patients. However, further studies in proper diabetic experimental models would be necessary.

It must be emphasized that action potentials changes were transient. We only were able to find an APD shortening during the IPoC maneuver (see Figure 1, Figure 5 and Figure 6). This would agree with data from Koletis et al. who was not able to find changes in monophasic action potential after 24 h of reperfusion [17]. This could be of clinical interest, as treatment with other antiarrhythmic agents have demonstrated that long term effects can be more dangerous than the potential acute benefits [43,44]. Future studies should clarify long term electrophysiological safety of IPoC.

We showed for the first time that IPoC induced a delay in both epicardial activation and in myocardial tissue resistivity recovery during reperfusion. As recovery of action potential amplitude was similar in all groups, the transient delay in epicardial activation is unlikely to be due to an alteration in Na^+^ currents. Alterations in passive electrical properties of the heart could explain the delay in both variables, and the antiarrhythmic effect of IPoC. Such alteration could modify the rapid spread of electrical activation required for the maintenance of reentrant circuits, involved in ventricular arrhythmias during the first critical minutes of reperfusion, and attenuate the proarrhythmic role of action potential shortening [45]. Myocardial passive electrical properties are dependent on both extracellular and intracellular characteristics, including intercellular communication through gap junction channels. In fact, our dye diffusion experiments showed reduced intercellular communication during IPoC (see Figure 2B). However, our results obtained in transgenic mice indicate that Cx43 is not essential for the observed delay in tissue resistivity recovery during reperfusion. The precise events responsible for those delays remain, thus, to be determined. Changes in extracellular edema (i.e., extracellular resistance) or in cell volumes (i.e., intracellular resistance) might explain, in part, these findings [46]. A reduction in tissue edema after IPoC was demonstrated by Zhao et al. [47]. Furthermore, the important contribution to impulse propagation of cell size as compared with gap junction number and distribution was previously emphasized by Spach et al. [48]. Finally, our results also confirm previous works demonstrating that, as occurred with the antiarrhythmic effect of IPoC during reperfusion, Cx43 is not involved in protection against infarction induced by the maneuver [49]. However, others have suggested that mitochondrial Cx43 might play a role in hypoxic postconditioning against infarction in H9c2 cells and neonatal rat cardiomyocytes [50]. These discrepancies deserve further research in the future.

Intermittent adenosine reproduced some of the IPoC electrophysiological changes, but with some side effects. We have demonstrated an anti-tachycardic effect and APD shortening, similar to the effects observed during IPoC. However, since ADOi doses used were high, the bradycardic effect was also pronounced. Interestingly, this bradycardia was limited to the drug exposure, but the antiarrhythmic protection persisted during reperfusion. However, in previous reports, intermittent adenosine during reperfusion failed to reduce infarct size [51]. Previous studies in pigs submitted to percutaneous coronary artery occlusion followed by reperfusion demonstrated lack of association between the antiarrhythmic effect of IPoC and changes in heart rate [4].

We are aware of some of the limitations of the present study. First, it was performed in normal juvenile rodents without preexisting atherosclerotic coronary plaques or myocardial dysfunction, which could affect myocardial responses to IPoC. Second, the involvement of adenosine receptors, PKC and KATP channels in IPoC has been demonstrated only with the use of pharmacological antagonists. However, our approach included carefully chosen concentrations based on published reports. Third, rats and mice are species that display short cardiac APD [37,52,53]. We only recorded action potentials from ventricular epicardial myocytes within the area submitted to ischemia and reperfusion. Thus, we cannot draw conclusions about what might be happening on the remaining surfaces, transmurally or in the endocardium. Caution should be taken when extrapolating the present data to other species.

## 4. Materials and Methods

The IAUC of the Facultad de Ciencias Médicas, Universidad Nacional de Cuyo (Protocol approval Nº 46/2015) and the Ethics Committee of the Vall d’Hebron Research Institute (CEEA 09/12), Universitat Autòmoma de Barcelona approved the present study. All procedures are in agreement with the National Institutes of Health Guide for the Care and Use of Laboratory Animals (NIH Pub. No. 85-23, Revised 1996).

### 4.1. Animal Models

Studies were performed in isolated hearts from Sprague Dawley rats and two transgenic mice strains. One strain was a knock-in in which the coding region of Cx43 is replaced by that of Cx32, and the other an inducible knock-out in which a marked deletion of Cx43 expression is obtained 14 days after 4-hydroxytamoxifen (3 mg/day suspended in vegetal oil, for 5 consecutive days) administration (Cx43^cre-ER(T)/flox^ mice) [53,54].

### 4.2. Experimental Protocols

Isolated rat hearts were submitted to 10 min of regional ischemia by occlusion of the left descending coronary artery. At the onset of reperfusion, hearts were randomly divided in IPoC group (*n* = 16), treated with 3 cycles of 30 s of reperfusion and 30 s of regional ischemia or Control group (*n* = 15). Adenosine receptor A_1_, A_2A_ and A3, PKC and K_ATP_ blockers or the corresponding vehicles (see below) were perfused simultaneously to the IPoC maneuver or alone during the first 3 min of reperfusion (*n* = 10/group).

Adenosine A_1_, A_2A_, or A_3_ receptor blockade was performed by adding to the perfusate cyclopentyl theophylline 10 µmol/L (CPT), SCH 58261 50 nmol/L (SCH), or MRS-1523 (MRS) 2 µmol/L, respectively. To inhibit PKC activity, chelerythrine (CHE, a cell-permeable non-selective PKC inhibitor, with half-maximal inhibition occurring at 0.66 µmol/L [55]. It inhibits α, β1, γ, and δ PKC isoforms) was administered at a final concentration of 2 µmol/L, whereas glibenclamide 10 µmol/L (GLI), was used as non-selective K_ATP_ channels inhibitor. SCH, MRS, CHE and GLI were dissolved in 0.25 mL/L dimethyl sulfoxide.

In additional hearts, adenosine 10 and 100 µmol/L were administered, intermittently after coronary flow restitution, in 3 cycles of 30 s of reperfusion and 30 s of adenosine (ADOi, *n* = 10/each dose). These treatments were compared with a group submitted to intermittent global ischemia as postconditioning stimuli (GIPoC, *n* = 10).

Mice hearts were submitted to 10 min of global ischemia and 10 min reperfusion for action potential studies (*n* = 5 each group) and to 30 min of global ischemia followed by 60 min reperfusion for impedance and infarct size measurements (n indicated below each column in Figure 4). In both cases, IPoC was induced by 6 cycles of reperfusion (10 s) and global ischemia (10 s). Mice hearts were continuously paced from the cardiac apex using rectangular pulses of 2.5 ms duration and 4V amplitude, at 133 ms basic cycle length.

### 4.3. Electrophysiological Studies

#### 4.3.1. Arrhythmias

Ventricular arrhythmias in isolated rat hearts were classified according to the Lambeth Convention and quantified using a score adapted from Curtis and Walker [52,56,57]. This score assigns a value to the severity of arrhythmias observed during every minute of reperfusion as follows: 0- Sinus Rhythm; 1- up to 10 premature ventricular beats; 2- more than 10 premature ventricular beats, bigeminy or salvos 3- non-sustained ventricular tachycardia (<30 s); and 4.- Sustained ventricular tachycardia (>30 s) or ventricular fibrillation.

#### 4.3.2. Electrograms and Action Potentials

Cardiac electrograms and transmembrane action potential’s from epicardial left ventricle cells, were obtained from rat hearts and from Cx43^flox/flox^ and Cx43^cre-ER(T)/flox^ mice, as previously described [23,52].

#### 4.3.3. Impedance

Myocardial electrical impedance and its two components, tissue resistivity and phase angle, were measured from rats and mice hearts using a four-electrode probe (interelectrode distance: 1 mm) placed in the left ventricle free wall at a frequency of the applied alternating current (10 μA) of 7 kHz, as also previously described [58].

### 4.4. Intercellular Chemical Communication

Chemical communication through gap junctional channels was assessed in isolated rat hearts, either submitted to IPoC or not after index ischemia (*n* = 4/group), by the Lucifer Yellow (LY)-Rhodamine (RD) diffusion assay, performed at minute 4 of reperfusion, as previously described [59].

### 4.5. Statistical Analysis

Data were expressed as mean ± SEM. Inferential analysis was performed using ANOVA or repeated measures ANOVA, followed by Bonferroni posttest, and contingency tables were treated by Fisher exact test, as appropriate. The duration of reperfusion arrhythmias was expressed as median and interquartile range (IQR) and analyzed using Kruskal-Wallis test followed by Dunn posttest. Mann-Whitney U test was used to assess differences in LY/RD ratio.

## 5. Conclusions

We conclude that the antiarrhythmic effect of IPoC is mediated by the release of endogenous adenosine that, acting through adenosine A_1_, A_2A_ and A_3_ receptors, induces shortening of the APD at the onset of reperfusion. Furthermore, whereas PKC activation is necessary for the protective effect of IPoC against reperfusion arrhythmias, but is not involved in APD shortening, K_ATP_ channels partially contribute to APD shortening, but not to the antiarrhythmic effect. Furthermore, the delay in both electrical activation and tissue resistivity recovery is not mediated by connexin 43. Changes in passive electrophysiological properties during IPoC deserve further investigation.

## Figures and Tables

**Table 1:** Duration of severe ventricular arrhythmias during reperfusion.

Experimental Groups	Ventricular Tachycardia	Ventricular Fibrillation
Control	45.0 (10–142)	476.0 (198–555)
IPoC	17.0 (3–146)	5.0 (0–115) **
CPT	11.0 (0–41)	459.0 (228–555)
IPoC + CPT	68.0 (28–235)	250.5 (121–291)
DMSO	45.0 (24–123)	529.0 (205–582)
IPoCd	27.5 (3–146)	10.5 (0–141) **
SCH	50.5 (33–110)	398.5 (124–460)
IPoC + SCH	64.0 (25–128)	282.0 (112–505)
MRS	35.0 (8–99)	520.0 (233–563)
IPoC + MRS	82.5 (20–186)	263.0 (0–415)
CHE	41.0 (20–51)	467.5 (295–545)
IPoC + CHE	71.5 (38–133)	360.0 (224–460)
GLI	401.0 (260–542) **	61.5 (18–75) *
IPoC + GLI	33.5 (12–63)	66.0 (12–90) *
ADOi10	141.0 (60–242)	91.5 (30–135) *
ADOi100	120.5 (22–195)	66.5 (0–106) *
GIPoC	39.5 (18–87)	48.0 (0–78) **

Values are expressed in seconds and correspond to median (interquartile range). Cyclopentyl theophylline (CPT), adenosine A_1_ receptor antagonist; dimethyl sulfoxide (DMSO), vehicle of the drugs; vehicle perfusion during IPoC procedure (IPoCd); SCH 58261(SCH), adenosine A_2A_ receptor antagonist; MRS-1523 (MRS), adenosine A_3_ receptor antagonist; chelerythrine (CHE), non-selective PKC inhibitor; glibenclamide (GLI), non-selective K_ATP_ channels inhibitor; adenosine intermittent (ADOi) during reperfusion; intermittent global myocardial ischemia as postconditioning stimuli (GIPoC). * *p* < 0.05 and ** *p* < 0.01 vs. control by Kruskal-Wallis.

## Abbreviations

APD	action potential duration
IPoC	ischemic postconditioning
PKC	protein kinase C
K_ATP_	ATP-dependent potassium channels
Cx32	connexin 32
Cx43	connexin 43
CPT	adenosine A_1_ receptor antagonist, cyclopentyl theophylline
SCH	adenosine A_2A_ receptor antagonist, SCH 58261
MRS	adenosine A_3_ receptor antagonist, MRS-1523
GLI	non selective K_ATP_ blocker, glibenclamide
LY	Licifer Yellow
RD	Rhodamine
